# *Acanthopanax senticosus* ameliorates steatohepatitis through HNF4 alpha pathway activation in mice

**DOI:** 10.1038/s41598-023-50625-z

**Published:** 2024-01-02

**Authors:** Yutaka Kawano, Maki Tanaka, Yasushi Satoh, Shigekazu Sugino, Jun Suzuki, Masaki Fujishima, Eri Okumura, Hideo Takekoshi, Osamu Uehara, Shintaro Sugita, Yoshihiro Abiko, Tetsu Tomonari, Hironori Tanaka, Hidekatsu Takeda, Tetsuji Takayama

**Affiliations:** 1 Department of Community Medicine and Medical Science, Tokushima University Graduate School of Biomedical Sciences, 3-18-15 Kuramoto-cho, Tokushima, Tokushima 770-0042 Japan; 2 Department of Gastroenterology and Oncology, Tokushima University Graduate School of Biomedical Sciences, Tokushima, 770-0042 Japan; 3 Department of Clinical Laboratory Science, School of Medical Technology, Health Sciences University of Hokkaido, Sapporo, Hokkaido 002-8072 Japan; 4 Department of Anesthesiology and Perioperative Medicine, Tohoku University School of Medicine, Sendai, Miyagi 980-8575 Japan; 5 Production and Development Department, Sun Chlorella Co., Ltd, Kyoto, 600-8177 Japan; 6 Division of Disease Control and Molecular Epidemiology, Department of Oral Growth and Development, School of Dentistry, Health Sciences University of Hokkaido, Tobetsu, Hokkaido 061-0293 Japan; 7 Department of Surgical Pathology, Sapporo Medical University School of Medicine, Sapporo, Hokkaido 060-8543 Japan; 8 Division of Oral Medicine and Pathology, Department of Human Biology and Pathophysiology, School of Dentistry, Health Sciences University of Hokkaido, Tobetsu, Hokkaido 061-0293 Japan; 9 Department of Physical Therapy, Sapporo Medical University School of Medicine, Sapporo, Hokkaido 060-8543 Japan

**Keywords:** Non-alcoholic steatohepatitis, Non-alcoholic fatty liver disease

## Abstract

Non-alcoholic fatty liver disease is a common liver disease worldwide, and is associated with dysregulation of lipid metabolism, leading to inflammation and fibrosis. *Acanthopanax senticosus* Harms (ASH) is widely used in traditional medicine as an adaptogen food. We examined the effect of ASH on steatohepatitis using a high-fat diet mouse model. Mice were fed a choline-deficient, l-amino acid-defined, high-fat diet with ASH extract (ASHE). After 6 weeks, liver RNA transcriptome sequencing (RNA-Seq) was performed, followed by Ingenuity Pathway Analysis (IPA). Our findings revealed that mice fed a high-fat diet with 5% ASHE exhibited significantly reduced liver steatosis. These mice also demonstrated alleviated inflammation and reduced fibrosis in the liver. IPA of RNA-Seq indicated that hepatocyte nuclear factor 4 alpha (HNF4 alpha), a transcription factor, was the activated upstream regulator (*P*-value 0.00155, z score = 2.413) in the liver of ASHE-fed mice. Adenosine triphosphate binding cassette transporter 8 and carboxylesterase 2, downstream targets of HNF4 alpha pathway, were upregulated. Finally, ASHE-treated HepG2 cells exposed to palmitate exhibited significantly decreased lipid droplet contents. Our study provides that ASHE can activate HNF4 alpha pathway and promote fat secretion from hepatocytes, thereby serving as a prophylactic treatment for steatohepatitis in mice.

## Introduction

Non-alcoholic fatty liver disease (NAFLD) has become a leading cause of chronic liver disease globally owing to lifestyle changes, such as food overconsumption and lack of exercise, causing obesity and diabetes^1^. In Japan, NAFLD prevalence has increased by 25.51% and is expected to affect almost half the population in 2040^2^. NAFLD is characterized by the overburden of fat in hepatocytes in the absence of other causes, such as alcohol intake and ranges from simple steatosis to non-alcoholic steatohepatitis (NASH). NASH causes inflammation and fibrosis, leading to liver cirrhosis and hepatocellular carcinoma, which could be life-threatening^3^. The global NASH prevalence could increase by 56%, and NAFLD mortality could double during 2016–2030^4^. Several drugs including sodium-glucose co-transporters, glucagon-like peptide-1 receptor agonists, peroxisome proliferator-activated receptor agonists, and fibroblast growth factor 21 analogs are clinically used in patients with NAFLD^5^. However, there is no definitive drug for preventing liver fibrosis progression in NAFLD patients. Therefore, exploring effective drugs that could intervene at early-stage NAFLD is required.

*Acanthopanax senticosus* (Rupr. et Maxim) Harms (ASH), also known as *Eleutherococcus senticosus* and Siberian ginseng, is a small hardy shrub native to China, Russia, Korea, and northern Japan^6^. Several food products and beverages containing ASH are available worldwide as a traditional Chinese medicinal herb, healthy botanical food, and adaptogen. ASH contains saponins, lignans, coumarins, flavones, and polysaccharides^7^, and possesses several pharmacological properties such as anti-oxidant^8^, -bacterial^9^, -allergic^10^, and -cancer effects on stomach cancer^11^, leukemia^12^, and hepatocellular carcinoma^13^. Iron overload and oxidative stress are known to causes liver dysfunction in some patients with NAFLD^14^ and hepatitis C virus infections^15^ as well as in mouse models^16^. However, the effect of ASH on NAFLD is still unclear. Therefore, we investigated the effect of ASH extract (ASHE) on liver steatohepatitis progression and explored whether ASHE could target specific genes and pathways.

## Results

### ASHE-containing high-fat diet-fed mice showed inhibition of progression of steatohepatitis

**Figure 1:**
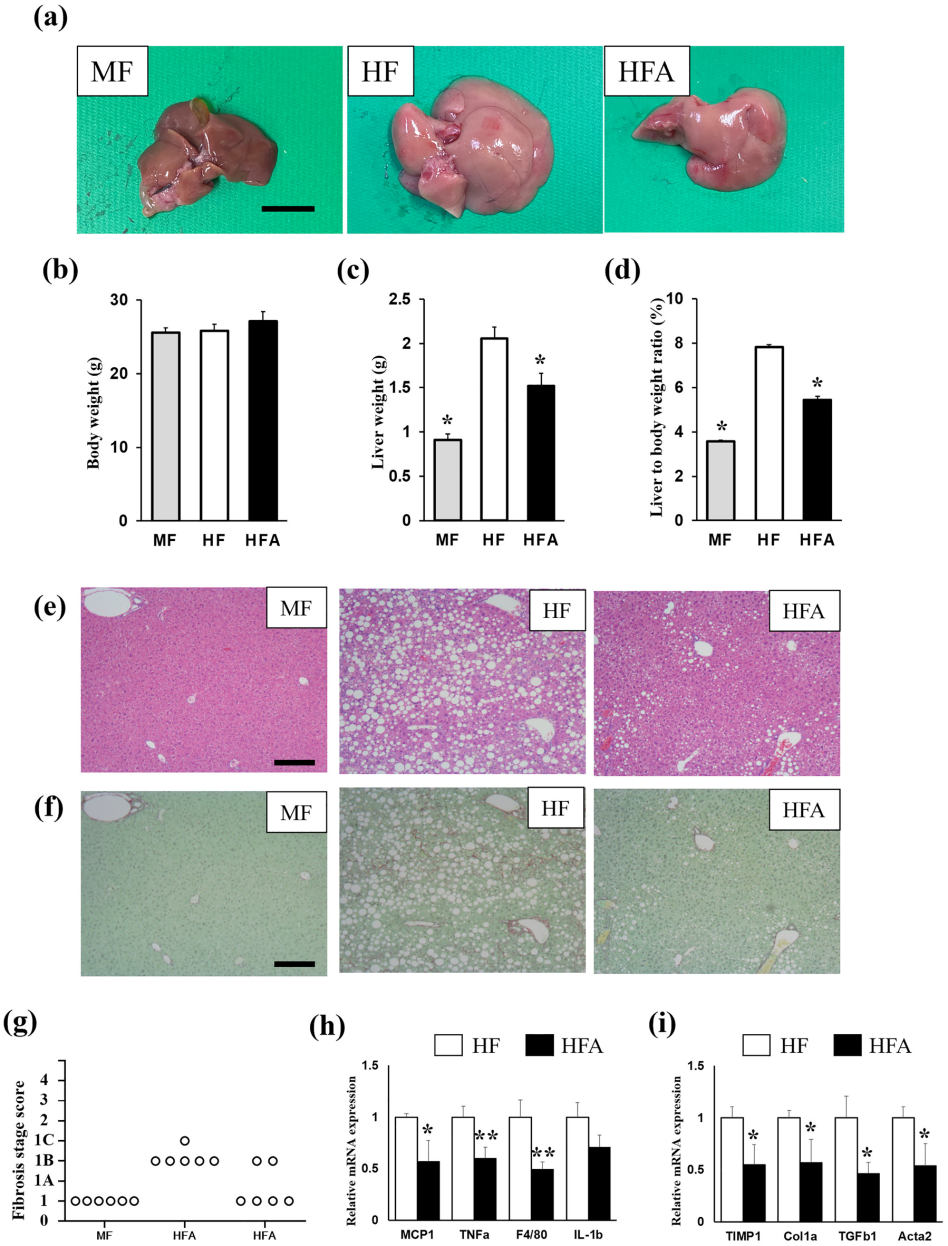
C57BL6/J mice were fed a regular diet (MF), or choline-deficient, l-amino acid-defined, high-fat diet (HF) with/without 5% ASHE (HFA) for 6 weeks. (**a**) Macroscopic images of the liver in each diet-fed mouse group. Scale bars, 10 mm. (**b**) Total body weight. (**c**) Liver weight. (**d**) Liver to body weight ratio (% body weight). *P < 0.001, statistical significance when compared to HF-fed mice. (**e**) Microscopic images of the liver stained by hematoxylin–eosin staining. (**f**) Microscopic images of the liver stained by Sirius Red-Fast Green staining. Scale bars, 200 µm. (**g**) Fibrosis stage score. (**h**) Gene expression of inflammation-related genes. (**i**) Gene expression of fibrosis-related genes. Data represent the mean ± SD (n = 6). *P < 0.05, **P < 0.01, statistical significance compared to HF-fed mice.

Male C57BL/6J mice (10 weeks old) were fed an MF, HF, or HFA diet for 6 weeks. The liver size of HFA-fed mice was macroscopically smaller than that of HF-fed mice (Fig. 1a), and the liver color in HFA-fed mice was less yellowish-white than in HF-fed mice. Whereas no significant difference in body weight was observed among the three groups (Fig. 1b), the liver weight of HFA-fed mice was significantly decreased compared with that of HFA-fed mice (Fig. 1c,d). The amount of food intake among the three groups was not significantly different (MF: 3.23 ± 0.53 g/mice/day, HF: 3.36 ± 0.20 g/mice/day HFA: 3.30 ± 0.16 g/mice/day) (Table 1).

**Table 1:** Characteristics of MF, HF, and HFA-fed mouse group.

	MF	HF	HFA
Body weight (g)	25.5 ± 0.7	25.7 ± 0.9	26.8 ± 1.1
Food intake (g/day)	3.23 ± 0.53	3.36 ± 0.20	3.30 ± 0.16
Liver weight (g)	0.9 ± 0.1*	2.0 ± 0.2	1.6 ± 0.2*
Liver weight ratio (%)	3.6 ± 0.3*	7.8 ± 0.5	5.4 ± 0.4*
Plasma TG (mg/dL)	36.6 ± 15.0	40.0 ± 12.5	28.3 ± 4.7
Plasma TC (mg/dL)	85.7 ± 8.7*	42.6 ± 7.2	96.6 ± 13.5*
Plasma AST (IU/L)	84.4 ± 12.6*	345.3 ± 86.2	230.7 ± 75.1
Plasma ALT (IU/L)	21.7 ± 6.6*	393.5 ± 31.5	253.6 ± 26.5*
Liver TG (mg/g liver)	67.7 ± 15.1*	314.1 ± 17.5	207.4 ± 75.4*
Liver TC (mg/g liver)	2.9 ± 0.2*	3.8 ± 0.2	3.1 ± 0.3*

Data are represented as mean ± SD, *P < 0.05, significant difference vs. HF fed mice (n = 6 per group).

Aspartate aminotransferase (AST) and ALT levels in plasma were increased in HF-fed mice. In contrast, HFA administration significantly decreased ALT levels, and the liver lipids of TG and cholesterol in HFA-fed mice decreased more than in HF-fed mice. Lipid droplets in hepatocytes were also less observed microscopically in HFA-fed mice (Fig. 1e). Histological features showed that the NAFLD activity score in HFA-fed mice (2.6 ± 0.8) was significantly lesser than that in HF-fed mice (5.4 ± 0.5), suggesting steatosis and lobular inflammation amelioration (Table 2). Sirius Red-Fast Green staining and histological features showed liver fibrosis development was suppressed in HFA-fed mice (Fig. 1f,g).

**Table 2:** Histological scores using the NAFLD activity score (NAS) on mice liver samples.

	MF	HF	HFA
Steatosis (score 0–3)	0	3	2.0 ± 0.6*
Lobular inflammation (score 0–3)	0	2.4 ± 0.5	0.6 ± 0.5*
NAS (score 0–8)	0	5.4 ± 0.5	2.6 ± 0.8*

NAS was scored as previously reported^33,34^. Data are represented as mean ± SD.

*P < 0.05, significant difference vs. HF fed mice. (n = 6 per group).

### Decreased inflammation and fibrosis-related gene expression in HFA-fed mice liver

To further examine steatohepatitis progression, the liver mRNA expression for inflammation and fibrosis-related genes was investigated in HF and HFA-fed mice (Fig. 1h,i). All four inflammation-related genes (Monocyte chemotactic protein 1; MCP1, Tumor necrosis factor-alpha; TNFa, F4/80 and IL-1b) except IL-1b were significantly reduced in HFA-fed mice compared with in HF-fed mice. The tissue inhibitor of metalloproteinase 1 (TIMP1), collagen 1 alpha (Col1a), transforming growth factor beta 1 (TGFb1), and actin alpha 2 (Acta2) genes were significantly decreased in HFA-fed mice. These results suggest that ASHE treatment might prevent lipid accumulation, inflammation, and fibrosis progression in the NASH mouse model.

### HNF4 alpha pathway activation in HFA-fed mice liver

**Figure 2:**
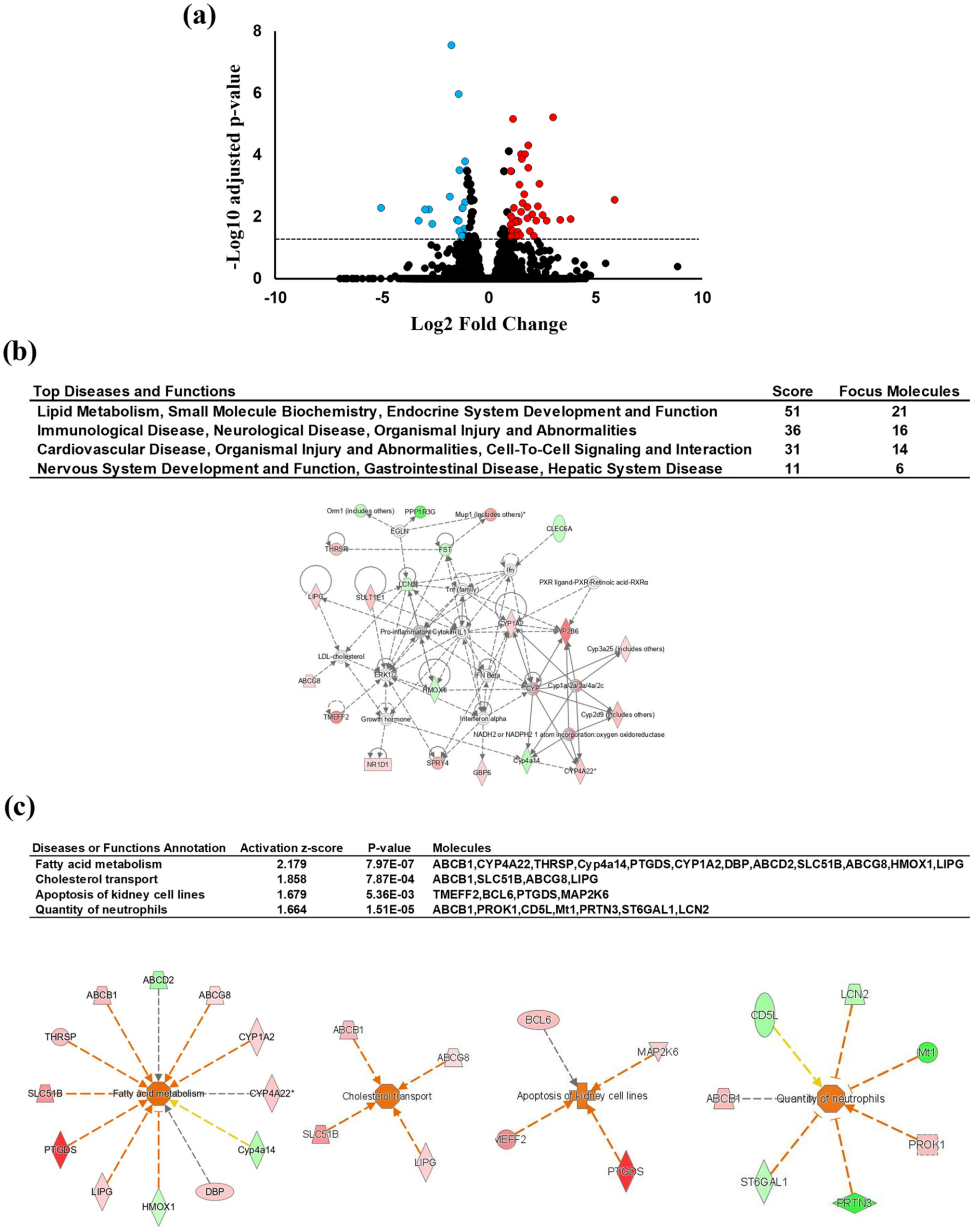
Results of liver RNA-Seq and IPA between HF and HFA-fed mice (n = 3). (**a**) Volcano plot of DEGs. The red circle indicates the genes with significantly increased expression (fold change more than 1 and adjusted *P*-value < 0.05) in HFA-fed mice compared with HF-fed mice. The blue circle indicates the genes with decreased expression (fold change less than − 1 and adjusted *P*-value < 0.05) in HFA-fed mice compared with in HF-fed mice. The horizontal line indicates the value of 1.30, corresponding to the adjusted *P*-value = 0.05. (**b**) Hepatic gene interaction network analysis in response to ASHE supplementation using IPA software. “Disease and functions” is the top four ranked-highest network. The score is based on the observed direction of target expression in the data set and the expected disease impact and function downstream indicated by the literature. The intensity of the node color indicates the magnitude of upregulation (red) or downregulation (green). Solid arrow: induction or activation or both; dashed arrow: suppression or inhibition or both. (**c**) Hepatic gene interaction and biological ontology analysis in response to ASHE supplementation using IPA software. “Disease and functions” annotation is the top-ranked highest ontology. The figure shows the “fatty acid metabolism” network with the highest activation z-score (2.179). The node color intensity indicates the magnitude of increased (red) or decreased gene expression (green). The orange arrow leads to activation, and the yellow arrow indicates findings inconsistent with the state of the downstream molecule, and gray arrow indicates the effect was not predicted. These images (**b**,**c**) were created using Ingenuity Pathway Analysis software (IPA, version 68752261; QIAGEN Inc., https://www.qiagenbioinformatics.com/products/ingenuity-pathway-analysis)^39^.

Global gene expression using RNA-Seq was conducted on HF and HFA-fed mice liver to elucidate the effect of ASHE on liver steatohepatitis. Thirty-eight DEGs were found to be statistically upregulated (fold change > 1 and adjusted *P*-value < 0.05), and 21 DEGs to be downregulated (fold change less than − 1 and adjusted *P*-value < 0.05) in the HFA group (Fig. 2a, Supplementary Table S1). Because ASHE includes some physiologically active ingredients that could enhance or negate its effect^17^, RNA-Seq interpretation summarizes integrated gene expression changes. Therefore, further analysis of these 59 DEGs was conducted using IPA. Figure 2b lists the network analysis constructed by DEGs as ranked by a higher score. The top four networks were as follows: “lipid metabolism, small molecule biochemistry, endocrine system development, and function”, “immunological disease, neurological disease, organismal injury, and abnormalities”, “cardiovascular disease, organismal injury and abnormalities, cell-to-cell signaling, and interaction”, and “nervous system development and function, gastrointestinal disease, hepatic system disease”. The top network had 21 focus molecules and the highest significance score of 51. Biological ontology, such as disease and functions classified by IPA, showed that these DEGs were associated with “fatty acid metabolism”, with the highest activation z-score (2.179) contributed to “cholesterol transport”, “apoptosis of kidney cell lines”, and “quantity of neutrophils” (Fig. 2c). These IPA data suggest ASHE could affect several genes related to lipid metabolism in the liver.

**Figure 3:**
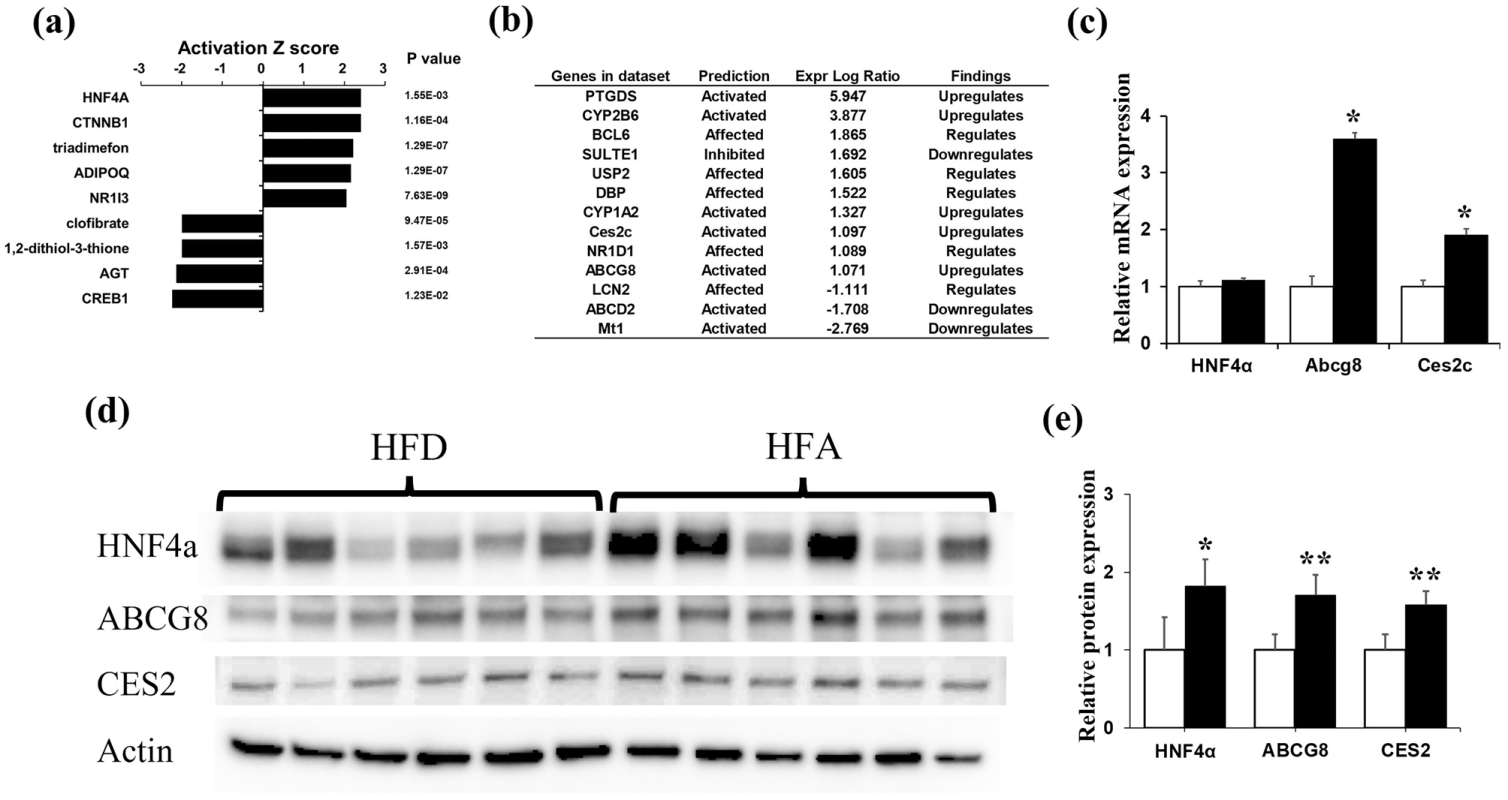
Upstream regulator analysis using IPA software. (**a**) The top nine upstream regulators along with the *P*-values are ranked by the magnitude of activation z-score. An activation z-score of > 2 indicates predicted to be activated, and less than − 2 is predicted to be inhibited. (**b**) The gene list for HNF4 alpha-target molecules in the dataset. (**c**) Quantitative RT-qPCR of mRNA gene expression levels of HNF4 alpha, ABCG8, and Ces2C. *P < 0.01, statistical significance when compared to HF-fed mice. (**d**,**e**) Western blotting of protein levels of HNF4 alpha, ABCG8, and CES2. Relative protein expression levels were calculated by normalizing the expression of each protein to that of mice actin. Cropped blots are shown and the original blots are available at the end of the Supplementary information. *P < 0.05, **P < 0.01, statistical significance compared to HF-fed mice.

We then explored the potential upstream regulators with which ASHE could affect these networks and biological ontology. Five gene pathways were statistically activated, and four were inhibited in HFA-fed mice (Fig. 3a). HNF4 alpha activation had the highest activation z-score of 2.413; seven (*PTGDS, CYP2B6, CYP1A2, Ces2c, ABCG8, ABCD2*, and *Mt1*) out of 13 genes had a measurement direction consistent with activation (Fig. 3b). HNF4 alpha is a transcription factor in the liver and could regulate some genes related to lipid metabolism^18^. Adenosine triphosphate (ATP)-binding cassette subfamily G 8 (ABCG8) and carboxylesterase 2 (CES2), whose expression is activated by IPA, were upregulated in our DEGs set data (Fig. 3b, Table S2).

We conducted qRT-PCR of *HNF4 alpha*, *ABCG8,* and *Ces2c* genes to verify whether IPA activates these genes and confirmed increased *ABCG8* and *Ces2c* gene expression in HFA-fed mice (Fig. 3c). Furthermore, western blotting showed that ABCG8, CES2, and HNF4 alpha protein expression increased in HFA-fed mice (Fig. 3d,e). These IPA analyses demonstrate that the HNF4 alpha pathway activation in the liver occurred in HFA-fed mice, followed by lipid accumulation inhibition in mice hepatocytes.

### Pre-treatment with ASHE decreases intracellular lipid contents caused by palmitic acid

**Figure 4:**
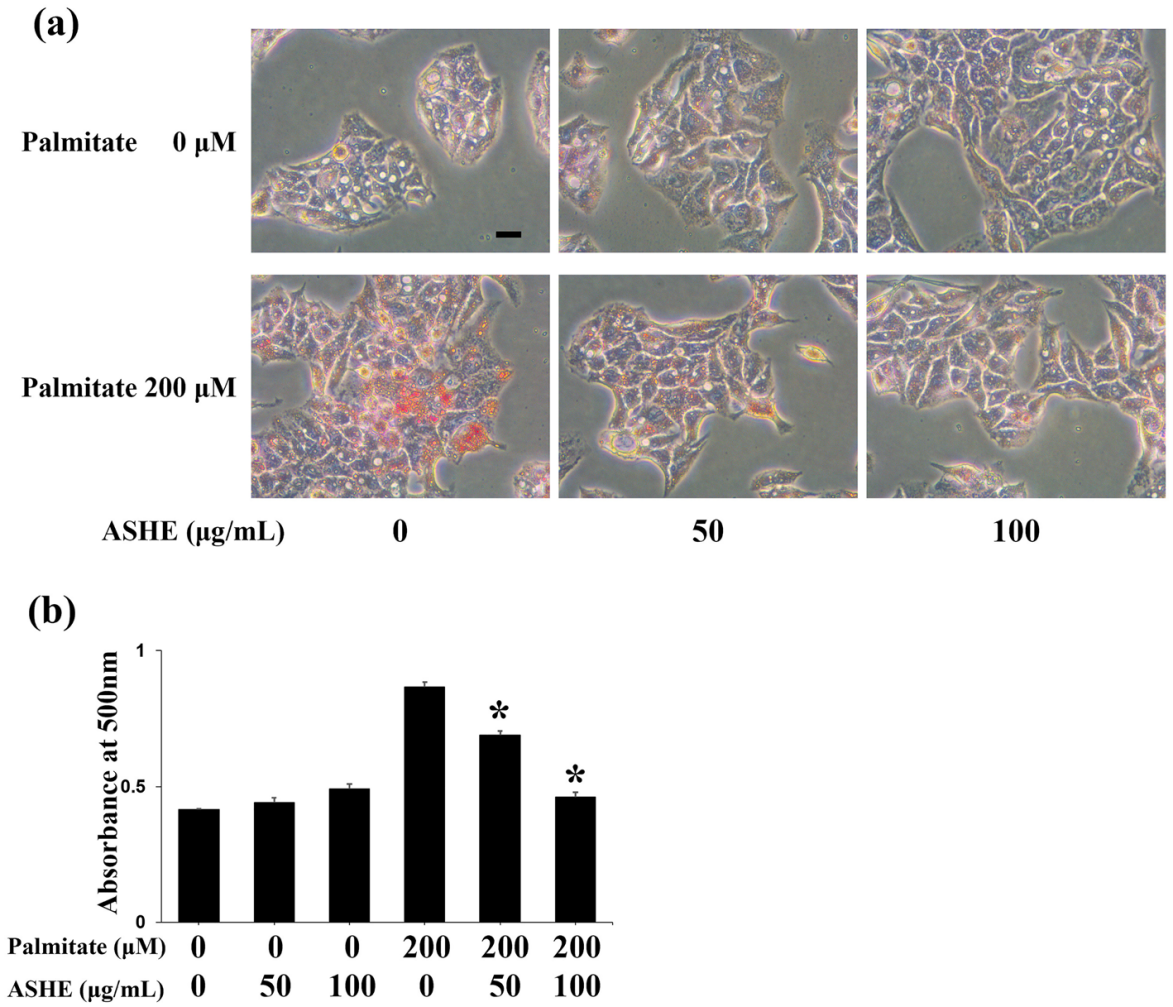
Intracellular lipid accumulation in palmitate-treated HepG2 cells. (**a**) Oil red O staining. Scale bars, 20 µm. (**b**) The extracted lipids stained with Oil red O were quantified by measuring the absorbance at 500 nm using a microplate reader. Data represent the mean ± SD (n = 3). *P < 0.01, statistical significance when compared to 200 µM palmitate.

The ABCG8 protein is a member of the subfamily of ATP-binding cassette transporters. In hepatocytes, ABCG8 promotes cholesterol and sterol secretion into the capillary bile duct^19^, and CES2 induces lipolysis in mice hepatocytes^20^. We then examined intracellular HepG2 cell lipid accumulation by treating cells with palmitic acid to determine whether increased expression of these proteins may decrease lipid content. Oil Red staining showed that pre-treating cells with 50 or 100 µg/ml ASHE for 48 h inhibited lipid accumulation in HepG2 cells (Fig. 4a,b). In summary, ASHE stimulates the HNF4 alpha pathway followed by ABCG8 and CES2 protein induction, decreasing lipid accumulation and inhibiting steatohepatitis progression.

## Discussion

We examined the effect of ASHE on fatty liver using a mouse model. Mice fed with ASHE containing HFA showed delayed steatohepatitis progression compared to mice fed with HF. The HNF4 alpha pathway was activated in HFA-fed mice liver, followed by induced ABCG8 and CES2 expression and preventing lipid droplets accumulation in hepatocytes.

HNF4 alpha is a nuclear hormone receptor highly expressed in the liver, intestines, pancreas, and kidneys^21^. HNF4 alpha in the liver promotes hepatocyte development and differentiation, affecting metabolic homeostasis^22^. Whereas HNF4 alpha gene knockdown increases liver lipid content, HNF4 alpha activation ameliorates steatohepatitis by modulating fat metabolism-related genes^23^. Our IPA results showed that liver DEGs between HF and HFA-fed mice accounts for lipid metabolism in network and fatty acid metabolism in biological ontology. Furthermore, these results also identified nine genes with upstream regulators, and HNF4 alpha had a highest activation z-score.

ABCG8 is a member of the ABC transporter superfamily. This ABCG8 protein in hepatocytes promotes cholesterol secretion into the bile duct, and mutations in this gene contribute to sterol accumulation and atherosclerosis^19^. CES2 is a member of the large carboxylase family and plays a role in thioester hydrolysis and transesterification. This protein also regulates fatty acyl and cholesterol ester metabolism^24^. CES2 attenuates steatohepatitis by inducing lipolysis in a mouse model^18,20^. In our study, upregulation of these genes activates HNF4 alpha as an upstream regulator in IPA.

Furthermore, the increased ABCG8 and CES2 expression verified a functional effect on lipid metabolism. Finally, an in vitro assay for lipid accumulation confirmed that ASHE pre-treatment decreased lipid contents in hepatocytes. Decreasing liver lipid content in HFA-fed mice was attributed to HNF4 alpha pathway activation, delaying fat deposition, inflammation, and fibrosis progression. Therefore, regular ASHE intake would be a promising method for steatohepatitis prophylaxis.

Most natural botanical extracts contain substantial compounds. However, high-performance liquid chromatography and mass spectrometry to identify these compounds are time-consuming and expensive. IPA is a web-based bioinformatics application that can analyze the gene expression profile using a built-in scientific literature-based database. Integrating the upstream regulatory factor analysis results with downstream effects could generate hypotheses about specific phenotypes and functions induced downstream from upstream events. When a specific pathway is identified using IPA during treatment with crude material, one could explain the pharmacological effect without isolating a specific compound. ASHE has 97 compounds^17^, including isoflaxidin, eleutheroside B, eleutheroside E, and chlorogenic acid, and none affects the HNF4 alpha pathway. Our study suggests that the effect of ASHE on HNF4 alpha activation may be due to an unknown material or a synergic effect.

Among the 59 DEGs identified using RNA-Seq (Supplementary Table S2), the expression of prostaglandin D2 synthase (*PTGDS*) gene was the most upregulated in the HFA group (log ratio: 5.946682494, adjusted P-value: 0.00993) and that of placenta-specific 9a (*Plac9a*) gene was the most downregulated in the HFA group (log ratio: − 5.018315556, adjusted P-value: 0.00532). PTGDS catalyzes the conversion of prostaglandin H2 to prostaglandin D2, which is known to have several pharmacological functions such as the development of allergy, asthma, and inflammation^25^. Furthermore, *PTGDS*-knockout mice exhibit not only insulin resistance but also increased visceral adipose tissues under high fat diet^26^. In our IPA analysis of annotation for diseases and functions (Fig. 2c), PTGDS was annotated for “fatty acid metabolism,” suggesting the positive effect of ASHE on lipid metabolism in mouse hepatocytes, but the detailed mechanisms remain unknown. Plac9a is highly expressed in the placenta and embryonic body^27^ and plays a role in extracellular matrix remodeling in heart-derived endothelial cells^28^. A recent study showed that plac9 inhibits the proliferation of human embryonic hepatocytes^29^. Although there is no study on the effect of ASHE on these two molecules, our results are informative in considering the systemic effects of ASHE on steatohepatitis when applied in the clinical settings.

However, this study had a limitation and only highlights the effects of ASHE on liver gene expression, and the effects of ASHE on other organs, such as fat tissue and intestines, may also be involved in these results. Therefore, whether the changes in liver gene expression caused by ASHE is direct or indirect remains unknown. Additionally, whether ASHE could ameliorate steatohepatitis should be elucidated in a human trial in the future.

In conclusion, ASHE administration improved steatohepatitis in a mouse model. HNF4 alpha pathway activation enhances ABCG8 and CES2 expression, decreasing hepatocyte lipid contents. ASHE is already available as a commercial food product, and regular intake could be a candidate for NASH prophylaxis.

## Methods

### Animals

Six-week-old male C57BL/6J mice were purchased from Sankyo Labo Service Corporation, Inc. (Tokyo, Japan). The mice were housed in groups of three per cage in a humidity and temperature-controlled room with a 12 h light/dark cycle. All animal experiments were approved by the Animal Experimental and Ethics Committee of the Health Sciences University of Hokkaido (permission number: 21-057) and performed in compliance with the guidelines of the Committee of Animal Care and Use of the Health Sciences University of Hokkaido and ARRIVE guidelines. The mice were allowed ad libitum access to their respective diets and distilled water. After acclimatization to a regular diet (MF: 12% kcal fat with a caloric value of 359.0 kcal/100 g (Oriental yeast CO., LTD Tokyo)) for 4 weeks, mice were divided into three groups according to the type of food intake as follows: (1) MF, (2) choline-deficient, l-amino acid-defined, a high-fat diet comprising 51% kcal fat with a caloric value of 461.2 kcal/100 g (HF: Oriental yeast CO., LTD Tokyo)^30,31^, and (3) HF supplemented with 5% ASHE (HFA) comprising 50% kcal fat with a caloric value of 455.6 kcal/100 g. The composition of each diet is shown in Table 3. A previous animal study determined the ASHE concentration (5%)^32^. After 6 weeks of feeding, mice were euthanized under anesthesia using isoflurane, followed by immediate blood and liver collection.

**Table 3:** Composition of MF, HF, and HFA diets.

	MF	HF	HFA
Protein	23.1	21.6	21.0
Lipids	5.1	26.4	25.1
Ash	5.8	2.6	2.9
Carbohydrate	55.3	34.2	36.4
Dietary fiber	2.8	6.2	5.9
Energy (kcal/100 g)	359.0	461.2	455.6

Each value indicates the amount in gram per 100 g.

*MF* regular diet, *HF* high-fat diet, *HFA* high-fat diet containing 5% ASHE.

### Blood biochemical analysis

Mice blood samples were collected from the inferior vena cava. After collection, serum was separated using centrifugation at 3000*g* at 25 °C for 10 min and stored at − 80 °C until use. Serum levels of alanine aminotransferase (ALT), total cholesterol, and triglyceride (TG) were examined at the Oriental yeast CO., LTD.

### Histological analysis

The mice liver tissues were fixed in Mildform (Fujifilm Wako Chemicals, Osaka, Japan), processed for paraffin embedding, and sectioned into 3 µm-thick sections. Each section was deparaffinized, rehydrated, and processed for hematoxylin–eosin and Sirius red-fast green staining. Sections were incubated in 0.04% Fast Green (Fujifilm Wako Chemicals), saturated in picric acid for 5 min, washed with distilled water, and incubated in 0.1% Sirius Red solution (Fujifilm Wako Chemicals) for 10 min. The histological features after each staining were determined using a Leica DMi8 microscope and LAS X version 3.3 (Leica Microsystems, Inc., Wetzlar, Germany). The steatosis, inflammation, and fibrosis degrees were assessed by an experienced pathologist blinded to the experimental procedures according to previously reported criteria^33,34^.

### Lipid analysis

Liver tissues were homogenized in chloroform/methanol (2:1, v/v), and lipid extracts were prepared using the Folch method^35^. Intrahepatic cholesterol and TG levels were measured using Cholestest CHO and Cholestest TG (Sekisui Medical, Tokyo, Japan), respectively.

### Liver isolation and total RNA extraction

Total RNA from mice liver was isolated using TRIzol^®^ reagent (Thermo Fisher Scientific, Inc., Waltham, MA, USA). The total RNA concentration was measured using a NanoDrop instrument (NanoDrop Technologies, Wilmington, DE, USA).

### RNA transcriptome sequencing and ingenuity pathway analysis

Complementary DNA (cDNA) libraries were prepared using Poly(A) messenger RNA (mRNA) Magnetic Isolation Module (New England Biolabs, Ipswich, MA, USA) and NEBNext^®^ Ultra™IIDirectional RNA Library Prep Kit (New England Biolabs) per the manufacturer's instructions to conduct RNA transcriptome sequencing (RNA-Seq). Pooled cDNA libraries were loaded onto a flow cell on an Illumina Novaseq 6000 sequencing system (Illumina Inc., San Diego, CA, USA), and 26 million paired-end reads (150 bp) were sequenced following the manufacturer’s instructions. The sequenced reads were aligned to the mouse reference genome (mm10) using HISAT2 version 2.1.0^36^. Read counts were calculated, and transcripts were assembled using featureCounts version 1.6.3^37^. The assembled transcript expression (fragments/kb of transcript/million mapped reads values) was compared between samples using DESeq2 version 1.24.0^38^.

Differentially expressed genes (DEGs) were determined using the likelihood ratio test. Significance was defined as results with an adjusted P-value of < 0.05 calculated using the Benjamini–Hochberg method to control the false discovery rate. DEGs were further analyzed using Ingenuity Pathway Analysis (IPA, version 68752261; QIAGEN Inc., https://www.qiagenbioinformatics.com/products/ingenuity-pathway-analysis)^39^. Upstream regulator analysis was performed within IPA, where potential upstream regulator activity is predicted based on the expression profile of known downstream targets to known upstream regulator-mediated expression changes reported in the literature. This analysis determines the significance of the detected targets overlapping with the upstream regulator through Fisher's exact test (P < 0.05) and implementing a z-score algorithm to predict the direction of upstream regulator activity change. A description of the z-score algorithm is available on the IPA website (http://www.ingenuity.com). RNA-Seq data used in this study are deposited and available in the DDBJ Sequenced Read Archive under the submission number DRA012226 (https://ddbj.nig.ac.jp/resource/sra-submission/DRA012226). Each accession number for sequence data is as follows; HF: DRR300717, DRR300718, DRR300719, and HFA: DRR300720, DRR300721, DRR300722.

### Quantitative reverse transcription‑polymerase chain reaction

cDNA was synthesized from 500 ng of total RNA using ReverTra Ace quantitative reverse transcription‑polymerase chain reaction (qPCR) RT Master Mix with gDNA Remover (Toyobo, Osaka, Japan) according to the manufacturer's protocol. qRT-PCR was performed in triplicate using KAPA SYBR FAST qPCR Master Mix (Kapa Biosystems, Wilmington, MA, USA) and CFX 96 Connect Real-Time System (Bio-Rad Laboratories Inc., Hercules, CA, USA). Thermocycling conditions were 3 min at 95 °C, followed by 40 cycles of 95 °C for 3 s and 60 °C for 20 s. Changes in relative gene expression between cDNA samples were determined using the 2^−ΔΔCq^ method^40^. The primers used in this study are listed in Supplementary Table S2.

### Western blotting

Western blotting was performed as previously described^41^. Briefly, snap-frozen liver samples were homogenized in a lysis buffer containing a protease inhibitor cocktail (Complete Mini, EDTA-free; Roche, Nutley, NJ, USA). Protein lysates (25 µg protein/lane) were loaded into 4–20% sodium dodecyl-sulfate polyacrylamide gel electrophoresis Mini-PROTEAN TGX precast gel (Bio-Rad Laboratories Inc.) and separated using electrophoresis (constant at 200 V for 30 min). The separated proteins were transferred to polyvinylidene difluoride membranes (Bio-Rad Laboratories Inc.) and blotted with specific primary antibodies overnight at 4 °C. The primary antibodies used were HNF4 alpha (1:1000; Santa Cruz Biotechnology, Inc., Dallas, TX, USA; cat. no. sc-374229), ABCG8 (1:2000; Abcam Inc. Cambridge, MA, USA; cat. No. ab223056), Ces2 (1:1000; Thermo Fisher Scientific, Inc.; cat. no. PA5-102415), and actin (1:3000; Santa Cruz Biotechnology, Inc.; cat. no. sc1615). After incubation for 1 h at room temperature with the secondary antibody, horseradish peroxidase‑conjugated anti‑rabbit IgG (1:2000; Cell Signaling Technology, Inc., Danvers, MA, USA; cat. no. 7074) or anti‑mouse IgG (1:2000; Cell Signaling Technology, Inc.; cat. no. 7076), signals were visualized using ECL Select Western Blotting Detection Reagent (GE Healthcare, Chicago, IL, USA). The bands were imaged using ChemiDoc XRS Plus (Bio‑Rad Laboratories, Inc.). Protein expression levels were quantified using ImageJ software 1.53 (National Institutes of Health), and relative expression levels were calculated from the expression ratio of each protein to mice actin.

### Lipid accumulation

HepG2 (JCRB1054) was obtained from the Japanese Collection of Research Bioresources and cultured in Dulbecco's Modified Eagle Medium (DMEM) supplemented with 10% fetal bovine serum, 10 mM l-glutamine, 100 U/ml penicillin, and 100 µg/ml streptomycin (Sigma-Aldrich; St. Louis, MO, USA) at 37 °C in a humidified incubator with 5% carbon dioxide. An in vitro lipid accumulation assay was conducted^42^. Palmitic acid (Sigma-Aldrich) was prepared by dissolving 195 mM solution in 100% ethanol, filtered, and stored at − 80 °C. This solution was then added to DMEM containing 10% fatty acid-free bovine serum albumin. For lipid accumulation induction, 2 × 10^5^ HepG2 cells were seeded in 12-well plates and treated with 200 µM palmitic acid. After 48 h of incubation, cells were fixed with 4% paraformaldehyde at room temperature for 1 h. Fixed cells were stained with 3 mg/ml Oil red O (Sigma-Aldrich) for 20 min, following staining with Mayer's hematoxylin solution (Fujifilm Wako Chemicals) for 1 min. After taking the representative images of stained cells using a microscope, intracellular lipids were dissolved using isopropanol (Fujifilm Wako Chemicals) for 5 min. The extracted lipids were then quantified by measuring absorbance at 500 nm using a microplate reader (ThermoScientific Multiskan FC, Thermo Fisher Scientific, Inc.).

### Statistical analyses

All data are presented as the mean ± standard deviation (SD). Differences between the two groups were assessed using the two-tailed unpaired Student's *t*-test, and differences among the three groups were calculated using a one-way ANOVA followed by a post hoc Tukey's test. All statistical analyses, except for RNA-Seq and IPA, were performed using SPSS version 21 (IBM Corp.).

### Supplementary Information

Supplementary Information.

## Data Availability

The data underlying this article are available on reasonable request to the corresponding author.
