# Supplementary material for: Acanthopanax senticosus ameliorates steatohepatitis through HNF4 alpha pathway activation in mice
Source: Sci Rep. 2024 Jan 2;14:110. doi: 10.1038/s41598-023-50625-z (PMC10762184; doi:10.1038/s41598-023-50625-z)

Supplementary information

Supplementary Table S1. The primer sequences (5'–3') used in this study for qRT-PCR

MCP1	F:	ATGCAGGTCCCTGTCATGCTTC
	R:	GGCATCACAGTCCGAGTCACAC
TNF alpha	F:	AGGGTCTGGGCCATAGAACT
	R:	CCACCACGCTCTTCTGTCTAC
F4/80	F:	GAAGGCTCCCAAGGATATGGA
	R:	TGCTTGGCATTGCTGTATCTG
IL-1b	F:	GGTCAAAGGTTTGGAAGCAG
	R:	TGTGAAATGCCACCTTTTGA
TIMP1	F:	AGGTGGTCTCGTTGATTTCT
	R:	GTAAGGCCTGTAGCTGTGCC
Col 1a	F:	TAGGACTGACCAAGGTGGCT
	R:	GGAACCTGGTTTCTTCTCACC
TGF b1	F:	CTCCCGTGGCTTCTAGTGC
	R:	GCCTTAGTTTGGACAGGATCTG
Acta2	F:	CCCGTGGTCATCGTCTCAG
	R:	GGACAGGGCACCATTGAAGG
HNF4 alpha	F:	CACGCGGAGGTCAAGCTAC
	R:	CCCAGAGATGGGAGAGGTGAT
ABCG8	F:	CTGTGGAATGGGACTGTACTTC
	R:	GTTGGACTGACCACTGTAGGT
Ces2c	F:	GCCAACCCCATCAGAAACACA
	R:	TTCAGCATGTCAAGATTTTGCAG
18S ribosome	F:	AGTCCCTGCCCTTTGTACACA
	R:	CGATCCGAGGGCCTCACTA

Supplementary Table S2. Differentially expressed genes (59) in the liver of HFA-fed mice compared to HF-fed mice.

ID	Symbol	Expr Log Ratio	Expr P-value	Expr False Discovery Rate (q-value)
ENSMUSG00000053168	9030619P08Rik	1.050132497	2.11E-05	9.93E-03
ENSMUSG00000040584	ABCB1	1.966583642	8.74E-05	2.92E-02
ENSMUSG00000055782	ABCD2	-1.708393761	8.21E-13	1.90E-08
ENSMUSG00000024254	ABCG8	1.070794203	5.15E-05	1.86E-02
ENSMUSG00000069170	ADGRV1	2.268584488	3.60E-05	1.41E-02
ENSMUSG00000024411	AQP4	1.535961108	1.41E-05	7.26E-03
ENSMUSG00000074794	ARRDC3	1.103159466	8.31E-05	2.82E-02
ENSMUSG00000022508	BCL6	1.864520818	1.45E-07	2.79E-04
ENSMUSG00000015854	CD5L	-1.341182353	1.91E-07	3.16E-04
ENSMUSG00000061825	Ces2c	1.096634714	2.66E-07	3.42E-04
ENSMUSG00000001128	CFP	-1.19413972	8.52E-06	5.27E-03
ENSMUSG00000030364	Clec2e/Clec2h	1.749863847	3.52E-08	9.82E-05
ENSMUSG00000023349	CLEC6A	-1.093727114	4.99E-06	3.60E-03
ENSMUSG00000032310	CYP1A2	1.327416164	1.55E-04	4.22E-02
ENSMUSG00000030483	CYP2B6	3.876652384	2.82E-05	1.25E-02
ENSMUSG00000096852	Cyp2d9 (includes others)	1.846693101	7.70E-06	5.08E-03
ENSMUSG00000061292	Cyp3a25 (includes others)	1.066023232	2.46E-07	3.42E-04

ENSMUSG00000028715	Cyp4a14	-1.445562446	2.95E-05	1.28E-02
ENSMUSG00000078597	CYP4A22	1.567665167	6.05E-08	1.40E-04
ENSMUSG00000059824	DBP	1.521933071	1.38E-04	4.12E-02
ENSMUSG00000034258	FLVCR2	-1.378087417	3.30E-05	1.36E-02
ENSMUSG00000021765	FST	-1.185677268	1.46E-04	4.22E-02
ENSMUSG00000033676	GABRB3	2.424885904	8.34E-07	8.76E-04
ENSMUSG00000092021	GBP6	1.267405836	4.20E-05	1.56E-02
ENSMUSG00000046167	GLDN	-2.609200138	4.70E-05	1.72E-02
ENSMUSG00000110151	Gm38416	2.065365834	1.72E-05	8.66E-03
ENSMUSG00000106069	Gm6135	2.560421724	1.84E-05	9.02E-03
ENSMUSG00000005413	HMOX1	-1.083035223	8.25E-08	1.73E-04
ENSMUSG00000034459	IFIT1B	1.169621236	9.44E-05	3.03E-02
ENSMUSG00000026822	LCN2	-1.110731163	1.42E-04	4.14E-02
ENSMUSG00000053846	LIPG	1.365056607	3.33E-05	1.36E-02
ENSMUSG00000020623	MAP2K6	1.405482404	1.03E-04	3.21E-02
ENSMUSG00000031765	Mt1	-2.768590222	1.02E-05	5.88E-03
ENSMUSG00000073830	Mup1 (includes others)	2.7377345	3.35E-05	1.36E-02
ENSMUSG00000028469	NPR2	1.25897042	1.01E-04	3.20E-02
ENSMUSG00000020889	NR1D1	1.089424706	1.81E-04	4.64E-02
ENSMUSG00000061540	Orm1 (includes others)	-1.361172326	1.45E-10	1.12E-06
ENSMUSG00000047617	PAXX	1.145315187	2.52E-05	1.16E-02

ENSMUSG00000094800	Plac9a	-5.018315556	8.99E-06	5.32E-03
ENSMUSG00000050423	PPP1R3G	-3.241620484	3.61E-05	1.41E-02
ENSMUSG00000070368	PROK1	1.846908518	2.62E-05	1.18E-02
ENSMUSG00000057729	PRTN3	-2.965458788	1.07E-05	6.01E-03
ENSMUSG00000015090	PTGDS	5.946682494	3.75E-06	2.94E-03
ENSMUSG00000053219	Raet1d/Raet1e	-1.773962543	2.71E-06	2.32E-03
ENSMUSG00000089809	RASGEF1B	-1.132064218	7.24E-05	2.50E-02
ENSMUSG00000097451	Rian	1.42185645	3.93E-05	1.49E-02
ENSMUSG00000108884	RP23-306P12.3	2.140985226	1.68E-04	4.42E-02
ENSMUSG00000026249	SERPINE2	1.455780612	1.00E-06	9.63E-04
ENSMUSG00000053862	SLC51B	3.042332638	1.09E-09	6.27E-06
ENSMUSG00000020027	SOCS2	1.204201187	8.43E-06	5.27E-03
ENSMUSG00000024427	SPRY4	2.314914733	6.98E-06	4.74E-03
ENSMUSG00000022885	ST6GAL1	-1.005122668	1.91E-07	3.16E-04
ENSMUSG00000029272	SULT1E1	1.691571636	2.24E-06	1.99E-03
ENSMUSG00000029553	TFEC	-1.343826518	9.41E-05	3.03E-02
ENSMUSG00000035686	THRSP	1.869408552	1.30E-08	4.98E-05
ENSMUSG00000026109	TMEFF2	3.396736161	3.15E-05	1.35E-02
ENSMUSG00000051682	Treml4	-1.182741011	8.68E-06	5.27E-03
ENSMUSG00000032010	USP2	1.604780387	5.49E-06	3.84E-03
ENSMUSG00000044206	VSIG4	-1.211180096	1.69E-04	4.42E-02

Uncropped western blots used in Figure 3d

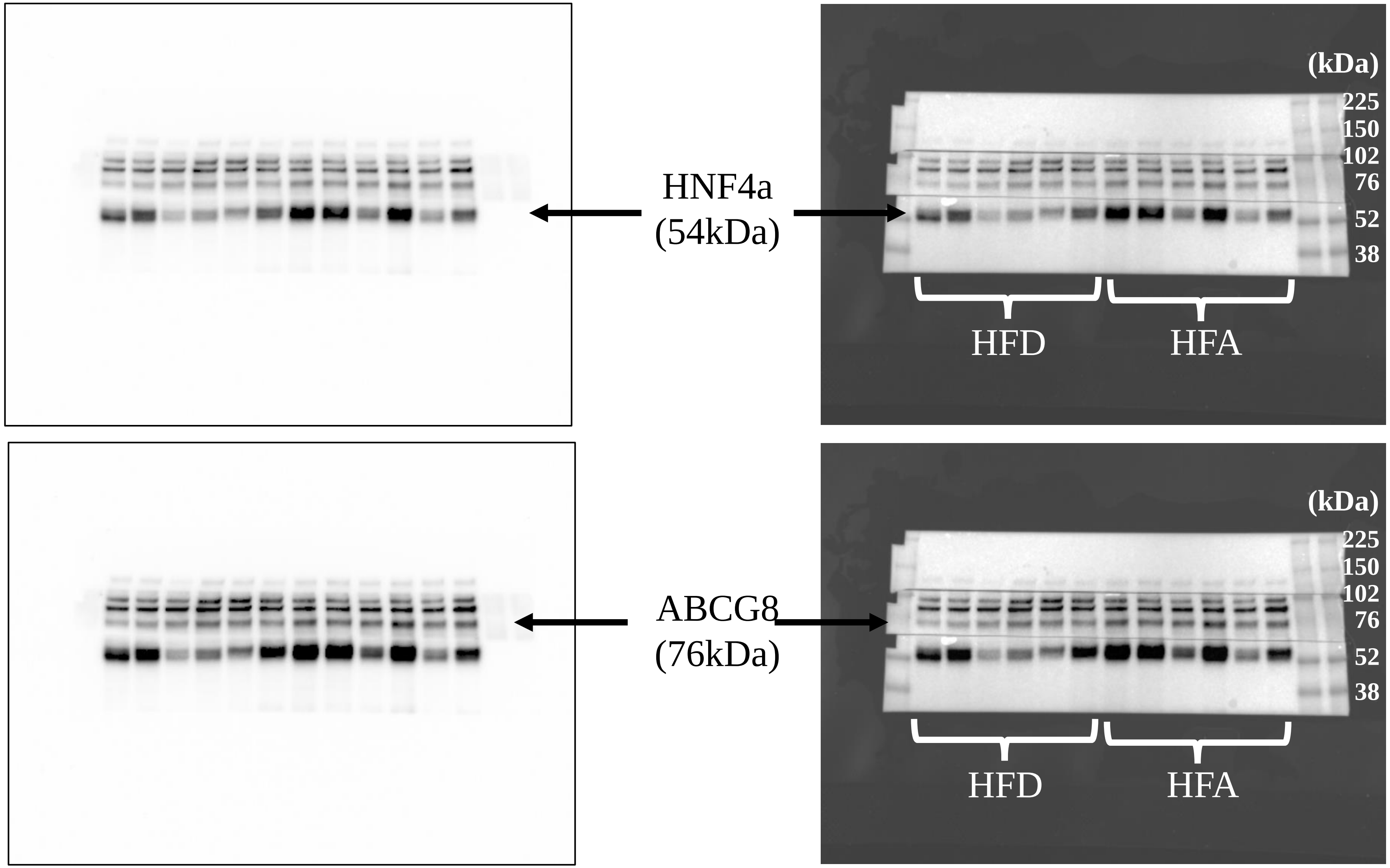

Uncropped western blots used in Figure 3d

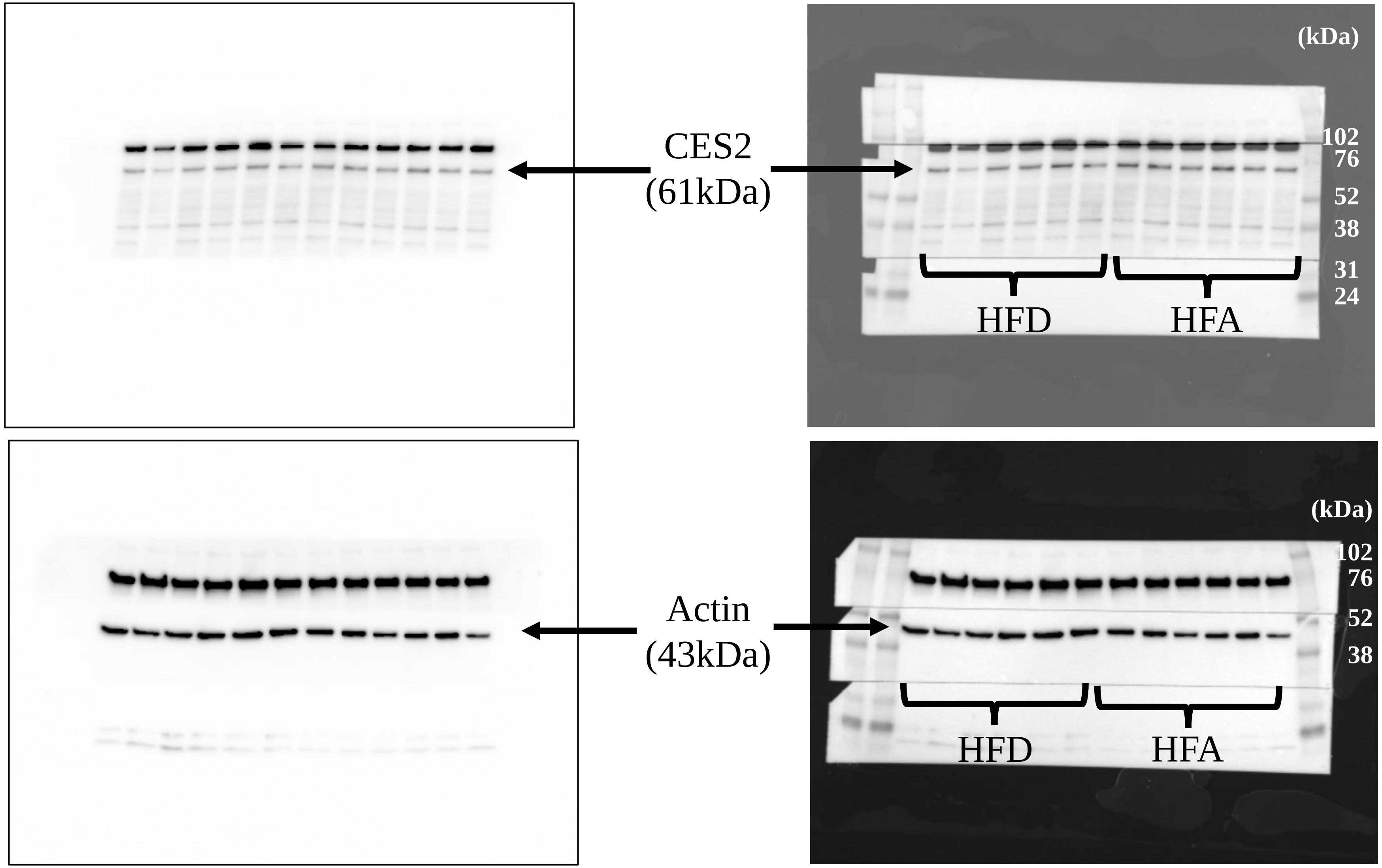

Supplement: Supplementary file 1 — Supplementary Information. [file 41598_2023_50625_MOESM1_ESM.pdf]
